# Anxious temperament and cyberchondria as mediated by fear of COVID-19 infection: A cross-sectional study

**DOI:** 10.1371/journal.pone.0255750

**Published:** 2021-08-05

**Authors:** Włodzimierz Oniszczenko

**Affiliations:** Faculty of Psychology, University of Warsaw, Warsaw, Poland; Osaka City University: Osaka Shiritsu Daigaku, JAPAN

## Abstract

This study’s main goal was to evaluate the association between anxious temperament and the fear of COVID-19-related self-infection and infection in loved ones (family members, friends, relatives) and cyberchondria. The sample consisted of 499 men and women aged between 18 and 72 who were gathered from the general population via an online recruitment platform. A numerical rating scale comprising 11 degrees of fear was used to assess participants’ COVID-19-related fear, and affective temperaments were evaluated using Akiskal’s Temperament Evaluation of the Memphis, Pisa, Paris and San Diego Autoquestionnaire (TEMPS-A) scales. Cyberchondria was assessed using McElroy and Shevlin’s Cyberchondria Severity Scale (CSS). Small to medium positive correlations were found between depressive, cyclothymic, irritable and anxious temperaments and cyberchondria and between depressive and anxious temperaments and COVID-19 fears. However, no correlation was observed between the hyperthymic temperament and cyberchondria. Cyberchondria positively correlated with both COVID-19 fears scales, though the correlation coefficients were medium. Based on the results of linear regression analysis, only anxious temperament and COVID-19 fear of self-infection were significant predictors of cyberchondria. The analysis also revealed a significant indirect effect of anxious temperament on cyberchondria through fear of COVID-19 self-infection as a mediator between anxious temperament and cyberchondria.

## Introduction

The COVID-19 pandemic has been a source of psychosocial distress resulting from medical, social and economic problems, as well as numerous instances of unverified information regarding virus spread [1]. Additionally, the spread of the COVID-19 pandemic and the countermeasures applied, especially social distancing and quarantine, have caused changes in mental health, primarily in terms of the development of depression and anxiety [2, 3]. Many authors have also pointed to the role of COVID-19-related fears of infecting oneself and others and the development of serious somatic changes, even leading to death, due to the infection [4–8]. According to Ahorsu et al. [4], fear is usually strongly associated with infectious diseases; COVID-19 is no exception, as the virus’s transmission is rapid, uncontrollable and associated with high mortality. On a related note, Yıldırım et al. [9] confirmed that fear of COVID-19 was a good predictor of depression and anxiety and served as the mediator between perceived risk and depression and anxiety, while resilience served as a buffer factor.

A critical factor associated with a population’s level of anxiety and depression in the COVID-19 pandemic may be affective temperaments [10], see also Vázquez & Gonda [11], for a detailed description of temperaments. Researchers consider affective temperaments (depressive, cyclothymic, hyperthymic, irritable, anxious) as stable across the human lifespan [12]. According to Rovai et al. [13], a high level of affective temperaments, except hyperthymic, relates to human susceptibility to life stressors, mood disorders, anxiety and depression, and somatic disorders. In contrast, the hyperthymic temperament serves as a protective buffer against stressors.

An anxious temperament may be of particular importance in the context of fear of COVID-19 infection and health anxiety and searching for health-related information on the Internet. An anxious temperament is related to worry and rumination, excessive uncertainty and vigilance, and constant mental and physical tension that can turn into somatic symptoms [11]. According to Michl et al. [14], the rumination associated with anxious temperament can be a crucial mediator between demanding events and depression and anxiety. In a recent study, Gonda et al. [15] showed that affective temperaments were associated with emotional reactivity and an individual’s response to strong stressful events, such as assault or serious injury that could lead to developing depression. The potential importance of affective temperaments (including anxious temperament) to psychological distress associated with the COVID-19 pandemic has also been confirmed by Moccia et al. [16].

The COVID-19 pandemic has been associated with a high level of uncertainty about individuals’ mental and physical health. Conceivably, uncertainty may increase the fear of infection, especially when rumination may intensify this process. Ouellet et al. [17] suggested that a low level of uncertainty tolerance may be a component of basic fear under threatening conditions. The threat to life and health related to the COVID-19 pandemic may cause real fear associated with self-infecting and loved ones’ (family members, friends, relatives) infection. Such fear can trigger irrational worry related to the infection and motivate a person to seek information about risk factors. Fear of infection is natural, but health anxiety can turn into symptoms of pathological COVID anxiety manifested in cyberchondria symptoms [18].

Therefore, people with a high level of anxious temperament who more strongly experience the fear of COVID-19 infection will plausibly also be more prone to intensively search for information on the threat to their health, manifesting in cyberchondria.

Starčević and Berle [19] described cyberchondria as the tendency to over-worry about an individual’s health condition and engaging in increased research on the Internet for medical information about symptoms, which may be accompanied by general health anxiety, doubts and compulsions to explain them. Many researchers [20–22] have suggested that cyberchondria involves on frequent online searches for health information that exacerbate anxiety. According to Hashemi et al. [23], fear of COVID-19 is also directly predicted by cyberchondria, confirming the assumption that excessive searching for information about one’s health adversely affects human mental health.

Anxiety sensitivity, uncertainty intolerance and obsessive-compulsive symptoms are crucial predictors of cyberchondria. Additionally, Starčević et al. [24] showed that cyberchondria is a construct independent of obsessive-compulsive disorder and intolerance of uncertainty. In their meta-analysis, McMullan et al. [25] confirmed a general relationship between health anxiety and searching the Internet for information as well as between health anxiety and cyberchondria. In turn, Wang et al. [26] pointed out that avoiding excessive searching for information about health on the Internet can be a protective factor in relation to somatic and mental health in the context of the COVID-19 pandemic.

According to Starčević et al. [27], a key factor contributing to the development of cyberchondria during the COVID-19 pandemic has been the considerable uncertainty, both psychosocial and medical, associated with the pandemic. Information overload, including an excess of varied and difficult to distinguish information about a disease, has intensified the feeling of danger and fear of disease and has increased cyberchondria [28]. Maftei and Holman [29] suggested that uncertainty related to the medical aspects of the COVID-19 pandemic and social isolation could increase health anxiety. Meanwhile, among young people, compared to older people, an essential component in the development of cyberchondria is neuroticism, a personality trait strongly related to fear. Notably, neuroticism is also associated with affective temperaments [30] and COVID-19 anxiety [31], indirectly confirming the significance of affective temperaments in the development of cyberchondria.

We summarise our rationale for research as follows. The COVID-19 pandemic is associated with a high level of uncertainty, health anxiety, and fear of COVID-19-related self-infection and infection in loved ones. Moreover, anxiety and the aforementioned fears may contribute to the development of cyberchondria. That said, little is known about the role of affective temperaments in the development of COVID-19-related fears and cyberchondria. Consequently, this study investigated the relationship between the anxious temperament and fear of COVID-19-related self-infection and loved ones’ infection and cyberchondria. A high level of anxious temperament was hypothesised to show a positive association with the aforementioned fears and cyberchondria. We also assumed that COVID-19-related fears play a significant role as a mediator between anxious temperament and cyberchondria.

## Materials and methods

### Participants

The study involved 499 White participants, including 435 women and 64 men, aged from 18 to 72 (M = 29.34; SD = 9.76). The only inclusion criterion was a minimum age of 18. Table 1 presents a detailed sociodemographic description of the study sample. All participants were volunteers, and no one was remunerated for participation.

**Table 1:** Sociodemographic variables in the studied sample (*N* = 499).

Variable	N *(*%*)*
Gender	
Male	64 (12.8)
Female	435 (87.2)
Age in years (M ± SD)	29.34 ± 9.76
Marital Status	
Married	133 (26.7)
Single	230 (46.1)
Informal relationship	122 (24.4)
Divorced	14 (2.8)
Education	
Elementary	30 (6.0)
Secondary	166 (33.3)
Higher Education	303 (60.7)
Place of Residence	
Village, up to 20 thousand residents	112 (22.4)
City 21 to 100 thousand residents	85 (17.0)
City 101 to 500 thousand residents	95 (19.0)
City over 500 thousand residents	207 (41.5)

The data were collected between December 2020 and March 2021 via online self-report questionnaires. All participants provided informed consent to participate after reading the rules of the study displayed on a computer screen. By starting to answer the questionnaire, they gave their consent to participate in the study on the given terms.

The research project, including the procedure described and all questionnaires used, was approved by the local Research Ethics Commission at the Faculty of Psychology, University of Warsaw (ref: 16-11-2020).

### Measures

The Polish version of the Temperament Evaluation of the Memphis, Pisa, Paris and San Diego Autoquestionnaire (TEMPS-A) [10, 32] was used to assess affective temperaments. The TEMPS-A is a self-reported instrument comprising 110 items (109 for men) with a yes/no response format scored 1–0. Higher total scores on the TEMPS-A scales indicate higher levels of the respective temperament. Cronbach’s alphas for the study sample were as follows: depressive α = .76, cyclothymic α = .84, hyperthymic α = .82, irritable α = .80 and anxious α = .88.

Cyberchondria was assessed using the Cyberchondria Severity Scale (CSS) [33, 34]. CSS is a self-reported instrument comprising 33 items using a 5-point Likert-scale (1 = never; 2 = rarely, 3 = sometimes, 4 = often, 5 = always) to assess the extent to which a given statement relates to the participant’s behaviour. The Polish version of CSS includes four subscales; Cronbach’s alphas for the study sample were as follows: Compulsion α = .90, Distress α = .93, Excessiveness α = .85, Reassurance α = .8. The sum of the subscales forms the general cyberchondria index (Cronbach’s α = .94). In this study, we only considered the overall score for cyberchondria.

An 11-point numerical rating scale was used to measure the intensity of fear of COVID-19 infection for oneself and loved ones (family members, friends, relatives). Each participant assessed the severity of fear they experienced at the time of testing by pointing to a number on a scale from 0 (I feel absolutely no fear) to 10 (I feel unimaginable fear). Thus, a higher total score on this scale indicated greater fear. The same scale was used to assess COVID-19 fear in regions of the United States [7] and the threat of coronavirus infection among cancer patients undergoing therapy in a Polish population [8]. Notably, 11-point numerical scales are also used to diagnose pain intensity [35], assess pain and tiredness among cancer patients [36], and describe the stress experienced in different groups of people [37].

### Statistical analysis

The statistical analysis was performed using IBM SPSS Statistics 27 [38]. Descriptive statistics and skewness and kurtosis values of the examined variables were calculated. The correlation analysis among the variables was performed using the Pearson product-moment coefficients. According to Cohen [39], an absolute value of r of .1 was classified as small, .3 as medium and .5 as large. The mediation analysis was performed using the PROCESS Model 4 macro for SPSS v. 3.5 [40] with the bootstrap method (5,000 sample draws, 95% confidence intervals) [41].

## Results

Table 1 displays the sociodemographic data of the studied sample.

Table 2 provides descriptive statistics, including skewness and kurtosis values, for analysed variables. All the data analysed in the study reached the criterion of compliance with the normal distribution.

**Table 2:** Descriptive statistics, skewness and kurtosis values for analysed variables in the whole sample (N = 499).

Variables	Mean	Standard deviation	Skewness	Kurtosis
1. Depressive temperament	.48	.20	.16	-.67
2. Cyclothymic temperament	.48	.23	.04	-.89
3. Hyperthymic temperament	.37	.21	.24	-.74
4. Irritable temperament	.34	.19	.37	-.42
5. Anxious temperament	.88	.37	.05	-.85
6. Cyberchondria	63.11	20.76	.84	.58
7. COVID-19 SI	4.28	2.41	.36	-.80
8. COVID-19 LOI	6.37	2.77	-.79	-.23

*Note*: COVID-19 SI–Fear of Self–Infection; COVID-19 LOI–Fear of Loved Ones’ Infection.

Table 3 presents the Pearson r correlation coefficients between analysed variables in the studied group.

**Table 3:** Pearson r correlation coefficients between analysed variables in the whole sample (N = 499).

Variables	1.	2.	3.	4.	5.	6.	7.
1. Depressive temperament	-						
2. Cyclothymic temperament	.46***	-					
3. Hyperthymic temperament	-61***	-.13**	-				
4. Irritable temperament	.38***	.66***	-.09*	-			
5. Anxious temperament	.86***	.58***	-.49***	.49***	-		
6. Cyberchondria	.15**	.21***	-.03	.19***	.27***	-	
7. COVID-19 SI	.17***	.08	-.12*	.03	.27***	.29***	-
8. COVID-19 LOI	.17***	.10*	-.12**	.04	.28***	.23***	.64***

*Note*: COVID-19 SI–Fear of Self–Infection; COVID-19 LOI–Fear of Loved Ones’ Infection

* *p* < .05

** *p* < .01

*** *p* < .001.

Small to medium positive correlations were found between depressive, cyclothymic, irritable and anxious temperaments and cyberchondria; however, no correlation was identified between hyperthymic temperament and cyberchondria. Small to medium positive correlations were also found between depressive and anxious temperaments and COVID-19 fears. Small negative correlations were found between the hyperthymic temperament and COVID-19 fears. A small positive correlation was found between the cyclothymic temperament and fear of loved ones’ infection, while no correlation was found between a cyclothymic temperament and fear of self-infection. Cyberchondria positively correlated with both COVID-19 fears scales; the correlation coefficients were medium.

Linear regression analysis was performed to determine the extent to which anxious temperament and COVID-19 fears could be considered predictors of cyberchondria. The results of the regression analysis are presented in Table 4.

**Table 4:** Linear regression analysis of anxious temperament, self-infection and COVID-19-related fear of loved ones’ infection as predictors of cyberchondria in the whole sample (N = 499) with variance inflation factor (VIF).

Variable	*B*	SE *B*	ß	Semi-partial Correlations	VIF	Tolerance
Anxious temperament	11.23	2.49	0.20***	0.19	1.100	.909
COVID-19 SI	1.87	0.48	0.22***	0.16	1.727	.579
COVID-19 LOI	0.28	0.42	0.04	0.03	1.742	.574

*** *p* < .001; SE (standard error).

Based on the linear regression analysis results, only anxious temperament and COVID-19 fear of self-infection were significant predictors of cyberchondria. This combination accounted for 12% of the variance of cyberchondria rating (adjusted R^2^ = 0.12, with an effect size of f^2^ = 0.14).

**Fig 1:**
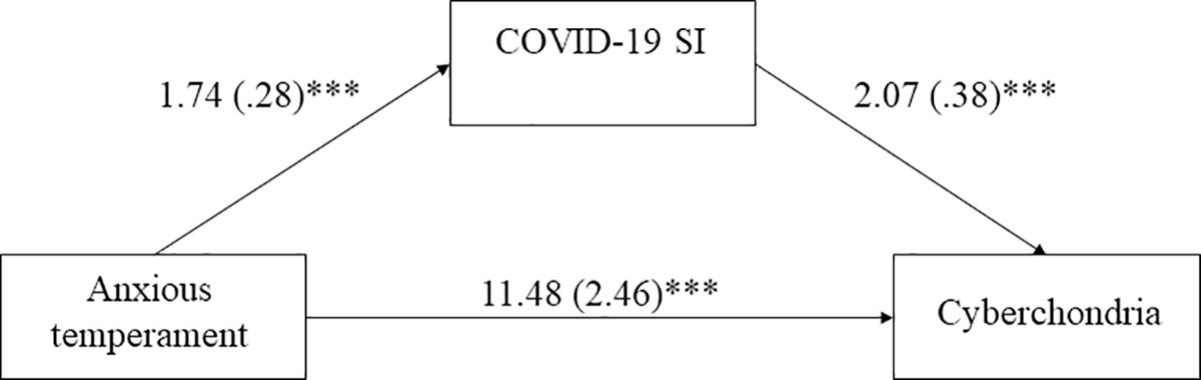
The mediating effect of COVID-19 SI in relationship between anxious temperament and cyberchondria. Unstandardised coefficients are reported, with standard errors in parentheses. *** p < .001.

In the last step, a mediation analysis with a bootstrapping procedure using COVID-19 fear of self-infection as the mediator between anxious temperament and cyberchondria was performed. The analysis revealed a significant indirect effect of anxious temperament on cyberchondria through COVID-19 SI [effect = 3.59, SE = .96, 95% CI = (1.88, 5.63)]. Fig 1 shows the individual pathways of the mediation analysis.

## Discussion

The study results confirmed a positive relationship between anxious temperament and cyberchondria as well as between anxious temperament and fear of COVID-19-related self-infection and loved ones’ infection, although the obtained correlation coefficients were medium. A positive relationship was also confirmed between cyberchondria and the mentioned fears. Again, the correlation coefficients were medium.

Akiskal [42] described anxious temperament as a chronic tendency to worry. On a related note, Starčević and Berle [19] indicated that the tendency to over-worry is an crucial component of cyberchondria. Thus, the tendency to worry is one element, though not the only one, linking the anxious temperament to cyberchondria. In one previous study, Vázquez and Gonda [11] indicated that anxious temperament is also characterised by hypervigilance and significant tension. If so, presumably, people with an intensely anxious temperament will be more focused on detecting disturbing changes in their own health and looking for medical information about symptoms. As indicated by Maftei and Holman [29], searching for information on the Internet may be a characteristic of younger people, for whom the Internet is a source of proven and unverified information on medical topics. The authors went on to suggest that neuroticism as a personality trait may play a key role in searching for information on the Internet in the young population. Soto [43] described individuals with a high level of neuroticism as “prone to experiencing anxiety, sadness, and mood swings” (p. 240). According to Brown et al. [44], although searching for information on the Internet can alleviate anxiety, this activity also causes a secondary increase in distress and triggers compulsive behaviour in subsequent searches for information. Notably, several studies have indicated a genetic correlation between neuroticism and obsessive-compulsive behaviour [45, 46]. Moreover, several authors [30, 47] have shown that neuroticism strongly correlates with anxious temperament, which may suggest that both of these features have a similar regulatory significance for behaviour, including cyberchondria.

Gonda et al. [15] suggested that anxious temperament might be of particular importance in situations involving human exposure to strong life stressors, such as diseases and injuries. In such conditions, anxious temperament turned out to be the only one among the affective temperaments that had a significant impact on the development of depression (i.e. one of the two most common consequences of the pandemic, apart from anxiety). Our study also showed a weak but significant relationship between a depressive temperament and cyberchondria, as well as between a depressive temperament and fear of COVID-19-related self-infection and infection in loved ones (see Table 2). This result may indirectly support the hypothesis about the importance of anxious temperament in developing cyberchondria.

The COVID-19 pandemic combines a highly contagious virus, high death toll and severe social limitations, all of which negatively affect human health in all its dimensions. One of the consequences of this influence is the development of fear of COVID-19-related self-infection and infection in loved ones (family members, friends, relatives). In our study, only the fear of COVID-19-related self-infection turned out to be a significant predictor of cyberchondria. In a similar vein, Coelho et al. [48] demonstrated that fear of COVID-19 was associated with hypochondria–an antecedent of cyberchondria–was related to obsessive-compulsive behaviour [19]. It is possible that in an emergency people will look for information primarily about themselves and their health. As Starčević [49] suggested, cyberchondria (hypochondria) may be associated with over-control. Obsessive-compulsive personality disorder may be associated with severe hypochondria and cause an excessive need for control, manifested by the perception of excessive threat to oneself, poor tolerance for uncertainty and distrust of oneself and others, along with a search for safety. Another hypothesis relates cyberchondria to the individual’s level of self-esteem. Along these lines, Bajcar and Babiak [50] showed that low levels of self-esteem in connection with health anxiety and obsessive-compulsive symptoms were factors in susceptibility to cyberchondria. Nevertheless, we have demonstrated the link between anxious temperament, COVID-19 fears and cyberchondria, providing an outline of the mechanism linking personality traits with cyberchondria.

The study has several limitations. First, the study participants were mostly women (men constituted only 12.8% of the sample). According to Vázquez and Gonda [11], affective temperaments have a characteristic gender-related distribution. Depressive and anxious temperaments predominate among women, while men tend to have a hyperthymic temperament. Therefore, the significantly larger proportion of women in our sample limits the possibilities of generalising the obtained results. Moreover, better-educated women living in large cities dominated the studied sample. Therefore, we recommend that future samples should include more men and should be more diversified in terms of education and place of residence. We also did not collect data on the current state of the participants’ health or their past illnesses because these data are legally protected. Nor did we control for other psychological variables, such as personality traits (e.g. neuroticism) or obsessive-compulsive behaviour, which could have been significant for the obtained results. Finally, we did not control the participants’ social situation, which might have influenced their condition at the time of the study.

## Conclusion

Despite these limitations, we demonstrated both direct and indirect relationships between anxious temperament and cyberchondria through fear of COVID-19-related self-infection. To our knowledge, this is the first study of its kind on the role of affective temperaments in cyberchondria.

## Supporting information

S1 Dataset (SAV)
